# Management of a pancreatic tail hydatid cyst: a case report

**DOI:** 10.11604/pamj.2023.45.124.40172

**Published:** 2023-07-14

**Authors:** Anis Hasnaouia, Racem Trigui, Sihem Heni, Houda Kammoun, Imen Sassi

**Affiliations:** 1 Department of General Surgery, Menzel Bourguiba Hospital, Menzel Bourguiba, Bizerte, Tunisia,; 2 Medical School of Tunis, Tunis El Manar University, Rue Djebel Lakhdar, Tunis, Tunisia,; 3 Department of Oncologic Surgery, Salah Azaiez Institute, Tunis, Tunisia

**Keywords:** Hydatid cyst, pancreatic tail, diabetes, conservative surgery, case report

## Abstract

The case report describes a 73-year-old woman, with a history of diabetes, who presented with left hypochondrium pain. Interrogation revealed a long-term history of living with Echinococcus granulosus endemic area, associated to close contact with sheep and dogs. Upon physical examination, a painless mass of the left hypochondrium, fixed to the deep plane. Abdominal ultrasonography (USG) showed a 9 cm encapsulated mass in contact with the tail of the pancreas. Further investigation was carried out by performing an abdominal computed tomography (CT) scan showing: large cystic mass with a partially calcified thickened wall, containing multiple vesicles, measuring 11.5 cm, located at the tail of the pancreas. The patient was put under Albendazole for a week and then operated on. During laparotomy, a hydatid cyst was located in the tail of the pancreas. Conservative treatment was done sparing the healthy pancreatic parenchyma and avoiding major surgery for a diabetic patient.

## Introduction

The hydatid cyst is a metacestode caused by the development in humans and many wild or domestic mammals of the larval form of a small dog taenia: *Echinococcus granulosus*. It is a frequent cosmopolitan parasitosis, especially in breeding areas. In Tunisia, it is rampant in a hyper-endemic mode and represents a major public health problem because of its high prevalence, the seriousness of its complications, and the significant economic losses it generates. For Hydatic cysts, the main localization is hepatic followed by pulmonary [1], but in particular circumstances, other rare sites may be reached, such as the pancreas. We present a case of primary hydatid disease of the pancreatic tail to put the focus on this rare entity and to insist on the importance and advantages of conservative surgical treatment if the diagnosis of hydatid disease is certain.

## Patient and observation

**Patient information:** a 73-year-old woman, with a history of diabetes, presented to our outpatient department for left hypochondrium pain evolving for two weeks, without pruritus, jaundice, or other associated signs. Interrogation revealed a long-term history of living in *E. Granulosus* endemic, associated with close contact with sheep and dogs.

**Clinical findings:** upon physical examination, a painless mass of the left hypochondrium, fixed to the deep plane, with no local inflammatory signs.

**Figure 1:**
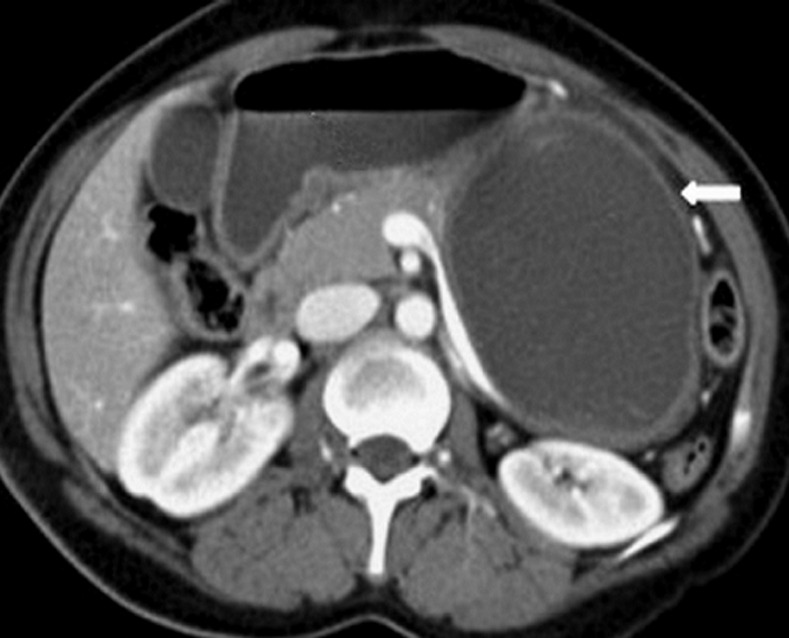
computed tomography scan of the hydatid cyst of the tail of the pancreas; large cystic mass (white thick arrow) measuring 11.5 cm, located in the tail of the pancreas

**Diagnostic assessment:** laboratory tests were within normal limits, especially with no eosinophilia. Serological tests were not performed for logistic reasons. Abdominal ultrasonography (USG) showed a 9-cm encapsulated mass in contact with the tail of the pancreas. Further investigation was carried out by performing an abdominal computed tomography (CT) scan showing: large cystic mass with a partially calcified thickened wall, containing multiple vesicles, measuring 11.5 cm, located in the tail of the pancreas (Figure 1).

**Diagnosis:** considering these findings, the diagnosis of a hydatic cyst of the pancreatic tail was retained. The patient was put under Albendazole for a week and then operated on.

**Figure 2:**
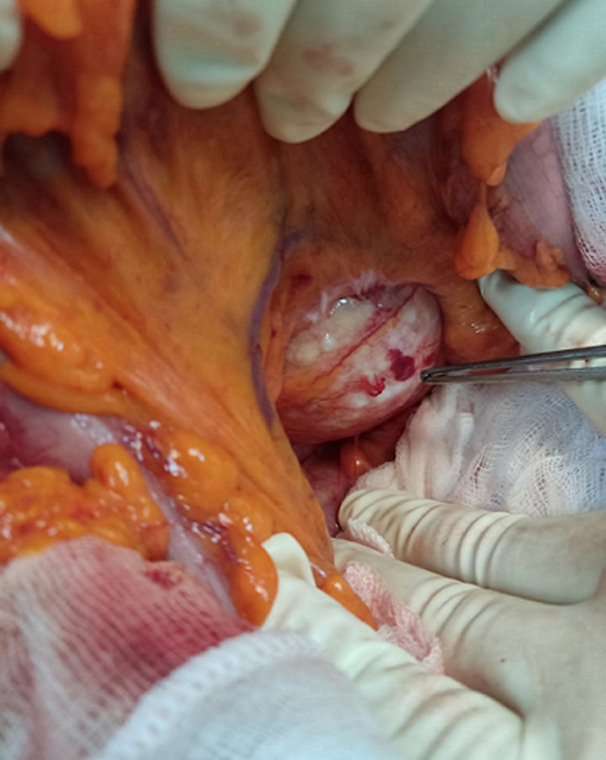
intraoperative view of the hydatid cyst of the tail of the pancreas; hydatid cyst of the tail of the pancreas pointed at with a dissecting forceps at the root of the transverse mesocolon

**Figure 3:**
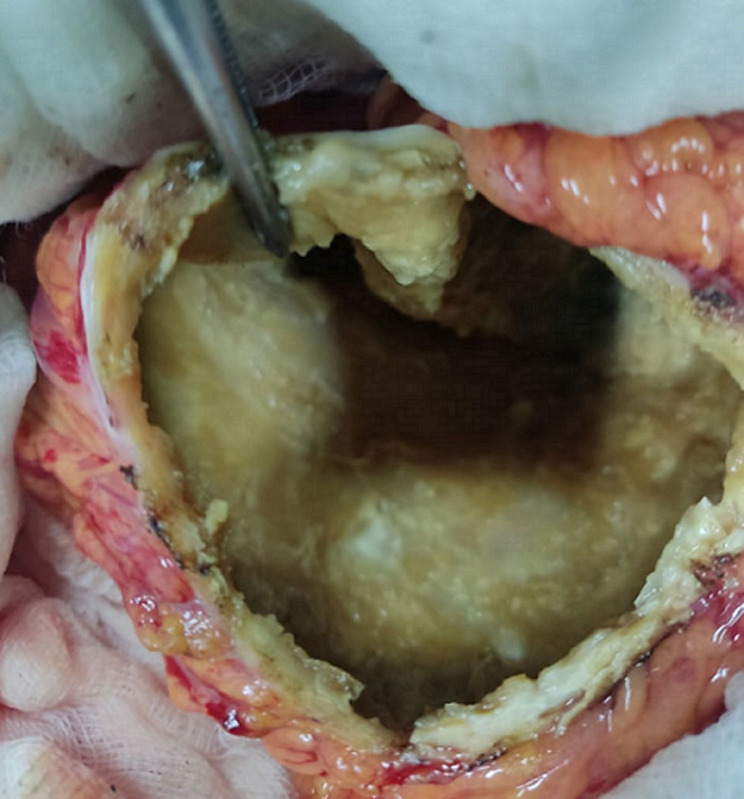
intraoperative view of the residual cavity; residual cavity of the hydatid cyst after the unroofing of the protruding dome

**Therapeutic interventions:** during laparotomy, a hydatid cyst was located in the tail of the pancreas (Figure 2). The cyst was fully aspirated bringing back hydatid gelatinous material. Sterilization of the cyst with hypertonic serum was performed followed by the unroofing of the protruding dome (Figure 3).

**Follow-up and outcome of interventions:** postoperative follow-up was uneventful, and the patient continued medical treatment based on Albendazole. Follow-up in 6 months showed no recurrence of hydatid disease.

**Informed consent:** a written consent was obtained from the patient to publish this case report.

## Discussion

The primary hydatid cyst (PHC) of the pancreas is a rare entity, accounting only for 0.1 to 2% of systemic echinococcosis [2]. The most common mode of pancreatic infestation is hematogenous when *E. Granulosus* tapeworm accesses the systemic circulation by crossing both hepatic and pulmonary barriers [3]. Other routes are also possible such as biliary dissemination when hydatic elements access the biliary tract and then into the pancreatic canal [4]. Lymphatic dissemination after cystic elements bypass intestinal mucosa accessing lymphatic channels by which they gain access to the pancreas known for its richness in lymphatic tissue. Veinous dissemination by immediate access to the pancreatic gland via the pancreatic veins, and last retroperitoneal dissemination. In most cases, the pancreatic hydatid disease is located in the pancreatic head (57%) due to its advanced blood supply, followed by the body (24%) and in some rare situations the tail (19%) [5]. Primary hydatid cyst is mainly asymptomatic, due to their slow growth rate estimated at 0.3 to 2 cm per year, therefore discovered accidentally or if complicated. Primary hydatid cyst of the pancreas can be challenging, not only because of the rarity of this entity but also because of its clinical presentation depending on so many variables (size, location, and complications) [6]. Hydatic cysts in the pancreatic tail, are most likely to remain asymptomatic until they reach enough size to press against adjacent structures or to be noticed clinically on inspection. Because of its rare incidence primary pancreatic hydatid disease is often misdiagnosed, but residency in areas where the hydatic disease is endemic, a life pattern with close contact with sheep or dogs, alongside a history of hydatic surgery are key elements that increase diagnostic yield, especially if associated to appropriate radiological and biological exams. The most executed imaging exams are USG, CT, and magnetic resonance imaging (MRI). Some cases require further investigation leading to the use of invasive tests such as endoscopic ultrasonography and endoscopic retrograde cholangiopancreatography [7]. Ultrasonography is the first-line exam for hydatic cysts, establishing their characteristics based on the World Health Organization (WHO) classification. The use of this classification in pancreatic hydatid cysts is less efficient than for liver hydatic disease, due to the peculiar retroperitoneal location of the pancreas and abdominal gas interposition. Computed tomography scan and MRI recognize a pancreatic cyst and determine its size, location, and relation to the wirsung duct. But the difficulty lies in linking this lesion to hydatid disease especially when the serology is negative. The pancreatic pseudocyst is distinguished from a cystic echinococcosis (CE) 1 hydatid cyst by the absence of an individualizable wall.

Cystadenoma and cystadenocarcinoma are distinguished from a CE 2 hydatid cyst by the enhancement of the edges and intra-cystic septa, after injection of contrast product. In the elderly, intraductal papillary mucinous neoplasms can take the same semiology as the hydatid cyst. The T2 sequences on MRI, the most sensitive examination, allow the differential diagnosis to be made. Other radiological signs can help to make the diagnosis of a pancreatic hydatid cyst: the presence of daughter cysts, an appearance of membrane detachment (water lily sign), or another abdominal or pulmonary hydatid location [8,9]. The presence of peri-cystic calcifications is suggestive of the diagnosis of hydatid cyst in an endemic area, but it must be kept in mind that these calcifications are not specific and can be found in cases of mucinous or even serous cystadenoma. To establish a positive diagnosis, patient screening, and surgery follow-up, serological tests are done using various techniques (enzyme-linked immunosorbent assay, indirect hemagglutination, serum immune-electrophoresis, complement fixation test, and immunofluorescence assay) [8]. However, it should be noted that the seropositivity rate is higher in liver hydatid disease compared to other sites of infestation and that seronegativity does not guarantee the absence of hydatic disease [9]. Surgery remains the cornerstone in the treatment of PHC of the pancreas. Multiple variables should be considered while keeping in mind that all levels of recommendations are low, corresponding to the opinions of experts. Choosing between conservative or radical management is not always an easy task. For PHC of the pancreatic tail: the tendency is toward radical surgery, especially if there is a fistula between the pancreatic duct and the cyst [10]. For our patient, as our country is endemic, as well as hydatid disease is always considered a benign disease, and to prevent morbidity and mortality of major surgery for an elderly patient suffering from diabetes, we decided to go with a conservative approach. This approach was the most sensible to preserve as much of the pancreatic parenchyma as possible and to avoid unbalancing her diabetes.

## Conclusion

We presented a compelling case of a pancreatic tail hydatid cyst, emphasizing the significance of conservative management in a patient with concomitant diabetes. Within this specific context, adopting a conservative approach becomes paramount whenever feasible. However, arriving at this decision is often fraught with challenges due to the potential resemblance of hydatid disease to other malignant lesions, such as mucinous cystadenomas of the pancreas, which commonly manifest in this anatomical region.
